# Ten simple rules for successful and sustainable African research collaborations

**DOI:** 10.1371/journal.pcbi.1012197

**Published:** 2024-06-27

**Authors:** Roseline Dzekem Dine, Lamis Yahia Mohamed Elkheir, Morufu Olalekan Raimi, Micheal Alemayehu, Salem Youssef Mohamed, Justice Kwadwo Turzin, Femi Qudus Arogundade, Elizabeth Akinyi Ochola, Alex Mukungu Nasiyo, Raziah Quallatein Mwawanga, Yahaya Abubakar Yabo

**Affiliations:** 1 Rinda Ubuzima, Kigali, Rwanda; 2 Department of Pharmaceutical Chemistry, Faculty of Pharmacy, University of Khartoum, Khartoum, Sudan; 3 Department of Environmental Management and Toxicology, Faculty of Sciences, Federal University Otuoke, Otuoke, Nigeria; 4 Department of Emergency and Critical Care, Tiruneshi Beijing General Hospital, Addis Ababa, Ethiopia; 5 Internal Medicine Department, Gastroenterology and Hepatology Unit, Zagazig University, Zagazig, Egypt; 6 Department of Biomedical Sciences, School of Allied Health Sciences, College of Health and Allied Sciences, University of Cape Coast, Cape Coast, Ghana; 7 Department of Non-communicable Diseases and Environmental Health, Public Health U–The Ulrich and Ruth Frank Foundation, Bethel, Minnesota, United States of America; 8 Centre for Global Health Research, Kenya Medical Research Institute (KEMRI), Kisumu, Kenya; 9 Department of Management Science, Project Monitoring and Evaluation, Kenyatta University, Nairobi, Kenya; 10 Discover Africa Thru Technology, Dar es Salaam, Tanzania; 11 Department of Veterinary Physiology and Biochemistry, Usmanu Danfodiyo University Sokoto, Sokoto, Nigeria; Carnegie Mellon University, UNITED STATES

## Introduction

Collaborative research consists of an equal partnership of researchers from diverse backgrounds who pursue mutually interesting and beneficial questions to achieve a common purpose via the coordination of activities and the sharing of knowledge, competencies, resources, and information, resulting in new scientific knowledge [1,2]. Hence, research collaboration could involve a continuum of organisational levels ranging from individual researchers to institutions, organisations, or even communities from different disciplines and geographical locations [1,3,4]. The quick expansion in international collaboration frameworks is primarily fostered by enhanced access to funding, sharing and standardisation of methodological expertise, and opportunities that increase the global impact and integrity of research findings [3,5,6]. Research outcomes from international cooperation contribute effectively to global stability, security, and prosperity. It also fosters equal opportunities by ensuring that communities are represented in global policies, particularly those related to health and sustainable development [7].

Historically, research within African countries was predominantly conducted by researchers from the Global North, often involving brief periods of fieldwork without establishing long-term collaborations [8]. However, African countries are currently experiencing a significant surge in both national and international collaborations, reflecting a shift towards more equitable and sustained research partnerships [9]. This change is driven by the continent’s unique research potential, offering opportunities not found elsewhere. To fully leverage these opportunities for global research and ensure African researchers’ contributions are recognised and integrated within the international scientific community, it is essential to outline key steps that foster effective partnerships. Recognising the evolving landscape of research in Africa, we propose solutions aimed at scaling meaningful collaboration with African researchers, ensuring mutual benefit and enhancing the impact of their work [10,11]. It is through such strategies that we can bridge historical gaps in collaboration and support a new era of inclusive and impactful scientific research.

## Rule 1: Understand Africa

Embarking on research within Africa requires an in-depth comprehension of its diverse geographical, social, economic, and political landscapes. Recognising Africa’s diversity is crucial, given its status as a continent with 54 United Nations-recognized countries, each distinct in culture, governance, and socioeconomic conditions, along with 2 independent states (Western Sahara and Somaliland) and several territories. This complexity is further nuanced by the region’s cultural diversity, which influences research methodologies, collaborations, and communication. To this end, understanding and managing cultural diversity effectively is beneficial and necessary for conducting meaningful research [12,13]. This understanding extends to recognising how local and regional conflicts, such as those in Tigray, Ethiopia, can significantly impact research endeavours. The effects of armed conflicts on the environment and indigenous farming systems, as highlighted by recent studies [14–17], underscore the need for researchers to be aware of and adapt to the realities on the ground. Additionally, North-South research collaborations, particularly in conflict and humanitarian contexts, face unique challenges and inequities that must be navigated with care and respect for all parties involved [15].

In light of these considerations, researchers are encouraged to engage deeply with the geographical, social, economic, and political contexts of their chosen research locales within Africa. This comprehensive understanding is fundamental to respecting local knowledge, identifying potential barriers, and possessing the requisite expertise to ensure research is conducted in a manner sensitive to local interests, needs, and priorities. Such an approach fosters sustainable development and guarantees that research activities are conducted ethically and equitably.

## Rule 2: Instil regular and open communication strategies

Effective research collaboration is enhanced by embracing and utilising a blend of communication strategies that combine the immediate access provided by virtual tools with the depth offered by in-person interactions. This multifaceted approach not only overcomes geographical and logistical hurdles but also enriches the collaborative process by integrating diverse cultural insights, thereby boosting mutual understanding and driving innovation across international teams [18]. By adopting robust communication strategies, collaborations address both practical and socio-emotional aspects, ensuring that all participants, irrespective of their geographical or cultural backgrounds, are fully engaged and contributing meaningfully to the project’s success. Effective and open communication is pivotal in cultivating a spirit of cooperation and fostering a culture of shared understanding and respect, which are essential for the success of international collaborations [19]. Virtual platforms such as Zoom, Microsoft Teams, Google Meet, etc. (summarized in Table 1) are indispensable for bridging geographical and logistical barriers, enabling researchers from varied locations to participate in discussions, share data, and collectively drive projects forward [20]. Moreover, virtual collaboration is extensively used to organise workshops, conferences, and seminars, hence, expanding participation opportunities beyond the usual travel and visa constraints. When managed effectively, virtual teams are efficient and reliable. Virtual communications yield productive results with reduced conflict, showcasing the potential of blended communication approaches to enhance the dynamics of research collaborations [20,21].

**Table 1:** A table summarising some open-source virtual tools that enhance communication and equitable access to resources among collaborators.

Category	Tools	Primary use
Communication	Zoom, Microsoft Teams, Skype, Google Meet WhatsApp, Slack (free versions)	Remote meetings, presentations, and training. Remote discussions and team interactions.
Project Management	Trello, Asana (basic free versions)	Organise tasks, timelines, and collaborative workflows.
Data Sharing, Storage and Management	Google Drive, Dropbox, WeTransfer, OneDrive (basic free versions) Zenodo	Store, share, and collaboratively edit research data and documents. Manage, share and preserve any research data in any size and format.
Writing and Publishing	Overleaf, Google Docs	Jointly write, edit, and publish research papers or reports.
Code Sharing and Revision	GitHub, GitLab, Google Colab Jupyter Notebooks Apache Zeppelin	Develop software, share and review codes. A cloud-based collaborative environment for machine learning, data analysis, and education. An interactive environment for live coding, equations, and visualisations. Notebook-based collaborative data exploration and visualisation.
Remote Access	TeamViewer, Chrome Remote Desktop OpenVPN, ProtonVPN (free versions)	Access remote computers or labs for research purposes. Securely access resources and ensure privacy in research collaborations.
Research Networking	ResearchGate, Academia.edu, Twitter(X), LinkedIn (basic features)	Connect with fellow researchers, discover research opportunities, and communicate your research.

Despite the advantages of virtual interactions, face-to-face interactions foster a level of collaboration fidelity that virtual tools have yet to match, particularly in environments rich in cultural nuances like Africa, where personal relationships significantly influence professional collaborations [20]. These interactions provide a unique space for spontaneous idea exchange, mentorship, and cultivating a shared vision and goals. Physical meetings also offer invaluable opportunities for cultural exchange and establishing deeper trust and understanding, crucial for fostering long-term partnerships and mutual respect. Acknowledging the challenges inherent in organising such meetings, including visa restrictions and logistical hurdles, underscores the need for meticulous planning, flexibility, and proactive communication with all stakeholders involved. Early coordination with collaborating organisations, conference organisers, and local governmental bodies can mitigate these challenges, ensuring meaningful participation from all collaborators.

Integrating hybrid models that combine virtual and physical participation can offer the best of both worlds, maximising engagement and adaptability. This approach is especially beneficial when travel is limited, or participants face scheduling conflicts. Ensuring open and transparent communication across all mediums is vital. Establishing a regular rhythm of updates and feedback is essential, allowing all team members to feel equally involved and valued. The culture of open communication should extend to decision-making processes, ensuring that all collaborators have an equal say in the direction and outcomes of the research. By embracing a dynamic and inclusive approach to communication, research collaborations can achieve greater depth, resilience, and productivity.

## Rule 3: Exchange research materials and resources responsibly

The disparity in resources between different regions of the globe is a significant barrier hindering research collaborations to tackle global challenges. African researchers often face substantial challenges due to limited or no funding, poor infrastructure, difficulties accessing advanced research materials and tools, and lack of databases and open data or software. This makes it challenging to conduct research and contribute to global scientific progress [22].

Exchanging valuable materials and tools between researchers worldwide offers a solution that provides both African researchers and their collaborators with access to resources not readily available to each other locally. The sharing of unique knowledge from the African continent enriches global research initiatives in areas like climate change, genotyping, sequencing, infectious diseases, and natural resource management [23]. Such collaboration not only fosters ethical and equitable partnerships but also ensures that African researchers are recognized as full partners, thus, safeguarding the rights and interests of all stakeholders involved in the projects. This approach deepens research impact and promotes mutual respect among international research communities.

Furthermore, collaborations promoting sustainable development should focus on addressing local needs, ensuring that benefits are equitably distributed and not solely favouring researchers from more developed regions. The Human Heredity and Health in Africa (H3Africa) initiative is a good example of this principle in action [24]. It spearheads health-related genomic research across Africa and supports the development of biorepositories in countries like Uganda, South Africa, and Nigeria through its affiliated network, H3ABioNet [25]. This infrastructure provides African researchers with essential resources and data and positions them to lead impactful research projects.

In addition to exchanging materials and tools, collaboration between African researchers and those from other parts of the world could involve joint research projects, mentorship programs, and training initiatives. These initiatives can help African researchers build skills and expertise while contributing to research projects of global importance. It is worth noting that in most cases, research projects with investigators outside the continent are held in high esteem and are published in prestigious journals.

## Rule 4: Support pan-African research

Collaboration among African countries remains limited, especially when compared to partnerships with more developed regions such as Europe, Asia, and America. This highlights a critical need for growth within the continent. Enhancing intra-African cooperation is vital as it offers a platform for sharing insights from diverse projects, leading to greater overall benefits and fostering sustainable relationships among projects, individuals, and organisations. Supporting pan-African research by investing in training and upgrading technology in research institutes across Africa would significantly improve research networking and collaboration. Given the continent’s diversity and the geographical proximity of its countries, there is a unique opportunity to address common challenges through collaborative research. Such cooperation can leverage similarities in disease prevalence, dietary habits, and cultural practices, facilitating easier movement of people and resources and driving collective advancement in African research and development.

The National Institutes of Health (NIH)-funded H3ABioNet consortium, a pan-African initiative, has significantly advanced skills in human genetics and sequencing across Africa [24]. This exemplifies how researchers and institutions within the continent could collaborate more effectively on similar projects, optimising the limited financial and material resources available. Such structured collaborative efforts not only enhance technical capabilities but also develop the soft skills essential for successful partnerships, as evidenced by programs like the Institute for Healthcare Improvement (IHI) and H3Africa.

Further exemplifying successful intra-continental collaboration is the partnership involving NIH, Wellcome Trust, the African Academy of Science (AAS), the Global Health: Science and Practice Journal, and H3Africa. This coalition has developed models and infrastructure that promote sustainable collaboration, equitable resource distribution, capacity building, and co-funding throughout Africa [25,26]. Additionally, initiatives like the African BioGenome Project (AfricaBP) aim to expand genomic and bioinformatics capabilities across the continent, demonstrating the vast potential for collaborative projects to build capacity and deliver tangible benefits for Africans [27].

For initiatives like these to thrive, it is crucial to secure increased support from African governments, regional organisations like the African Union, and international partners, including foreign governments, institutions, and foundations. Enhanced collaboration among African researchers and institutions can lead to a deeper understanding of the unique challenges presented by diseases such as COVID-19 within the African context. Such insights are essential for transforming the research landscape of African institutions. With improved research capabilities, African researchers can contribute more effectively to global efforts, such as vaccine research studies, ensuring vaccine development and distribution are conducted sustainably, justly, and equitably.

## Rule 5: Promote equity and inclusion

Promoting equity and inclusion in collaborative research in Africa is crucial for addressing the complex challenges faced by the continent. Research collaborations that leverage diverse expertise and resources are essential to effectively inform policy and practices. However, achieving this requires that all participants have equal access to opportunities and resources, enabling them to contribute meaningfully regardless of their diverse backgrounds or levels of experience. This would value each collaborator’s input and enrich research outcomes with various perspectives, leading to more innovative and comprehensive solutions to complex issues. Thus, fostering an equitable and inclusive environment in research collaborations is indispensable for leveraging the full spectrum of ideas and achieving groundbreaking advancements.

The Cape Town Statement, with its 20 recommendations for ethical research, underscores the necessity of embedding fairness, equity, and the acknowledgment of indigenous knowledge within the research ecosystem [28]. This initiative aims to correct the imbalance where, typically, high-income countries benefit disproportionately compared to their low- and middle-income counterparts in terms of authorship, career progression, and control over research priorities and outputs [29]. For instance, a recent study found that a significant portion of COVID-19 research related to Africa featured non-African authors taking the lead, spotlighting the urgent need for greater local involvement in scientific publications. This situation underscores the broader necessity for journals to actively incorporate local perspectives, particularly in research that impacts regions like Africa, thereby advancing equity and fairness [30]. Hence, the Cape Town framework aims to create a more equitable research environment by ensuring fair participation and acknowledgment across all research stages.

Despite these guidelines, challenges persist. Notably, the unequal distribution of resources and the need for low- and middle-income countries to prioritise research funding to reduce reliance on high-income country donations [31]. Cultural and linguistic diversity, while enriching, can also pose barriers to inclusive collaboration due to differences in communication, beliefs, and research practices [32]. Overcoming these obstacles is crucial for fostering a research landscape where equity and inclusion are at the forefront, enhancing the quality and impact of collaborative research efforts across Africa.

Cultural norms and hierarchical structures, particularly in Africa, can significantly influence power dynamics within research collaborations, which can stifle innovation by limiting diverse perspectives. This is often seen in the reverence for elders and authority figures, which might discourage younger researchers, women, or individuals from lower socioeconomic backgrounds from voicing their opinions or challenging established ideas. Encouraging participation across career stages—from early-career scientists to seasoned experts—and fostering a mentorship culture can help cultivate a more vibrant and inclusive scientific community. Moreover, cultural differences across Africa can affect how research is conducted and interpreted. For instance, some cultures may prioritise privacy, influencing research methodologies and data analysis, while others may favour openness, affecting the transparency and sharing of research findings. Addressing these cultural variances is crucial for promoting equity and inclusion within collaborations [33]. Developing research methods, communication strategies, and training programs sensitive to cultural nuances can ensure that research collaborations are more inclusive and equitable.

By establishing partnerships based on shared values like mutual respect, trust, and social justice and actively embracing diversity, research collaborations in Africa can become more inclusive and equitable. This approach not only enriches the research process but also ensures that the innovations and outcomes derived from these collaborations are more robust and reflective of the diverse perspectives across the continent.

## Rule 6: Incorporate a capacity-building component

Emphasising capacity building is fundamental for fostering equitable and effective research collaborations. This entails not just the personal development of individuals through training and mentorship but also strengthening institutional capabilities by enhancing access to the latest knowledge, advanced equipment, and essential resources. To ensure comprehensive capacity building, every research collaboration proposal must include a detailed plan on how these aspects will be developed and supported.

Beyond individual skill enhancement, capacity building encompasses staff exchanges that provide valuable exposure to diverse research environments and methodologies [34]. These exchanges, facilitated through partnerships between institutions and supported by international research bodies, are instrumental in transferring critical knowledge and expertise across regions. They also serve to forge lasting relationships among researchers from varied backgrounds, laying the groundwork for sustained collaboration and mutual learning.

To truly realise the benefits of capacity building, funding applications must explicitly outline strategies for improving individual skills, institutional resources, and overall research infrastructure. This includes ensuring access to cutting-edge technology, updating research methodologies, and securing the materials necessary for groundbreaking research. By strategically allocating resources and funding, we can establish sustainable collaborations that are mutually beneficial and geared towards achieving long-lasting impact and relevance in the scientific community.

Incorporating a comprehensive capacity-building component into research collaborations, as recommended by Haelewaters and colleagues [2], is indispensable for advancing equitable, inclusive, and impactful research endeavours. This approach elevates the research process and contributes significantly to the global body of knowledge, ensuring a brighter future for African research and beyond.

## Rule 7: Follow standard publication guidelines

Clear and transparent guidelines for publishing collaborative research are essential for fostering meaningful collaboration with African researchers. Such guidelines can help ensure that all researchers know their roles and responsibilities and can help promote fair and equitable authorship. Before starting a collaboration, researchers should develop a publication policy outlining authorship criteria, authors’ order, and each author’s responsibilities. The publication policy should be communicated to all researchers involved in the collaboration, including students, postdocs, and other trainees. It should be clear that all researchers must follow the policy, and violations should not be tolerated. This proactive approach prevents misunderstandings and conflicts by clarifying contributions and authorship order from the outset to ensure that publications resulting from such collaborations give credit to all contributors fairly, equitably, and respectfully.

Throughout the collaboration, all researchers should communicate openly and transparently about their research findings, data generated, and analyses. Any disagreements or concerns should be addressed early to avoid conflicts later in the process. A robust data management plan is also vital, detailing how data will be collected, analysed, stored, and shared, ideally in a secure, accessible, open-source location for transparency and future reference (Table 1).

Additionally, it is crucial to consider the strategic policies and regulations of each collaborating entity, such as organisation, nation, group, or country, and to align these with the overall goals and values of the project. Early discussions with collaborators about the collaboration’s objectives, responsibilities, and values foster a foundation of trust and mutual respect, which are necessary for sustainable and equitable partnerships. This strategic alignment and accountability are particularly important in ensuring that collaborations do not merely serve external interests but genuinely promote and enhance the involvement of African researchers, addressing the historical underrepresentation of researchers from developing countries in scientific publications [35]. This accountability should also extend to study participants to ensure that they are treated fairly and provided with all the details of the study and properly acknowledged in all publications [36].

## Rule 8: Make a data-sharing and management plan

Open and transparent data-sharing and management plans are crucial in building trust in research collaborations, especially when working across different cultures and regions where there may be differences in research practices and ethical standards. Sharing data can also help improve research quality by allowing for greater scrutiny and validation of results. This is especially important when researching complex and interdisciplinary topics involving multiple datasets and methods. Therefore, all collaborators should write and agree upon a data management plan with clear guidelines and protocols for data sharing and ownership. This is key to ensuring that research findings are protected, easily retrieved, and shared over the long term.

An effective data management and sharing plan should adhere to the Findable, Accessible, Interoperable, Reusable (FAIR) principle, ensuring that generated data is handled with high standards of accessibility and usability [37]. Thus, the data management plan must outline the types of data to be shared, the methods of sharing these data, and each collaborator’s specific roles and responsibilities in managing the data. The plan should also address the consequences of any breaches, such as the unauthorised sharing of participants’ data.

To ensure that the data is shared securely and ethically, the plan must comply with relevant data protection regulations and ethical standards. This includes utilising the appropriate data-sharing platforms and tools, such as secure data-sharing platforms and repositories, to safeguard the data effectively (Table 1). Moreover, universities and funders are encouraged to support Open Data initiatives, facilitating easier access to data for African researchers. This is needed to give equal rights and opportunities to promote accountable and sustainable research for every researcher.

## Rule 9: Consider local ethical approval policies

For decades, Africa has been a pivotal site for global research and discovery, significantly contributing to advancements across various fields. However, this role has occasionally led to exploitative practices, prompting a significant reevaluation of ethical research standards. This concern has highlighted the need for stringent ethical practices in collaborations between African researchers and their collaborating partners. To address these issues, specific local ethical standards and guidelines have been developed to ensure responsible research practices within Africa. These guidelines are designed to be rigorously adhered to by all researchers, including both local and international collaborators, to foster a culture of integrity and respect in research.

Ethical procedures are crucial for thoroughly evaluating researchers’ work, ensuring that conflicts of interest are minimised and the interests of study participants or subjects are protected. Research Ethics Committees (RECs) play a key role in this process by rigorously reviewing essential documents such as study consent forms, assent forms, and study protocols. These evaluations focus on assessing the risks and benefits of research, as well as ensuring voluntary participation, thereby reducing the potential for exploitation, among other ethical concerns [38,39].

Local ethical guidelines play a crucial role in building trust within communities historically affected by the adverse outcomes of failed clinical trials. These guidelines mandate that research participants receive thorough education about the study in their local languages, ensuring they are well-informed and protected from exploitation. Additionally, local ethical review boards often require the involvement of local investigators in research projects, which not only fosters ownership and builds local capacity but also promotes a respectful and ethical scientific environment. This involvement helps to prevent practices such as “helicopter” or “parachute” research, where external researchers conduct studies without adequate local participation or benefit to the community [38]. Furthermore, these measures aim to enhance the representation and impact of African researchers in scientific publications. A revealing statistic from cardiovascular research in sub-Saharan Africa shows that only 10% of native publishers are from the region, with the majority of publications authored by researchers from Europe, North America, and South Africa [40]. This highlights the critical need to prioritise local ethical approval policies to ensure that research activities are not only compliant but also respectful and beneficial to local communities. Adhering to these policies is essential for fostering trust and sustainable engagement within African research collaborations.

## Rule 10: Adhere to open science policies

In the pursuit of enhancing the reach and impact of African research collaborations, it is vital to support open science practices. For instance, publishing in open-access journals and depositing generated data in open-access repositories ensures that research findings are freely available, increasing their visibility and usage across the global scientific community. This approach not only democratises access to information but also encourages the integration of diverse scholarly contributions. Nevertheless, the costs associated with publishing in open-access journals can be prohibitive, particularly for researchers in low- and middle-income African countries. Therefore, it is important to include costs associated with open-access publishing in the research grant applications. This will reduce the barriers that may prevent researchers from publishing their work openly. Alongside these strategies, the promotion of preprints is essential as sharing preprints enables researchers to disseminate their findings rapidly and freely, accelerating the pace at which knowledge is exchanged and feedback is received from the broader community.

Furthermore, storing the generated data and protocols in publicly accessible repositories aligns with the FAIR principles, ensuring that datasets are findable, accessible, interoperable, and reusable. This practice facilitates the replication of research findings and enhances the transparency and credibility of scientific inquiries while strengthening the global research community’s ability to address complex scientific questions [37].

## Concluding remarks

Collaborative research is a pivotal tool for enhancing Africa’s scientific prowess, addressing the limitations of its national scientific systems, and bridging resource and infrastructure gaps [9]. Non-African countries also stand to gain from Africa’s unique research opportunities [11]. For instance, Africa carries the largest global burden of communicable infections, and with the nature of modern mobility across continents, infections are no longer confined to Africa but are becoming a global threat to public health [41]. Furthermore, Africa is also witnessing a considerable increase in the prevalence of non-communicable diseases, which are common among non-African societies but are driven by similar risk factors. The continent’s rich genetic diversity, holding the oldest and most diverse genome, is an invaluable resource for collaborative research. Such partnerships can lead to groundbreaking discoveries in understanding complex disease patterns and advancing precision medicine worldwide [41,42]. Investing in quality cooperative research in Africa represents a strategic investment in global disease prevention and treatment policies [11].

Apart from the wide range of potential benefits to global health systems, collaborative research in Africa could also provide substantial global socioeconomic merits. This is because the African population is the youngest and fastest growing globally, with sub-Saharan African countries estimated to account for more than half of the world’s population growth rate between 2019 and 2050. As a result, making an intellectual investment in this growing demographic is important in harnessing and growing talent that will lead the generations of the future [11]. Furthermore, as Africa continues to offer a critical mass of future consumer markets, developed products and technologies must be adapted to suit their needs and culture, which can only be achieved via collaborative research [11].

In conclusion, these 10 rules offer a comprehensive framework for fostering effective and sustainable research collaborations across Africa. From understanding Africa’s unique cultural and geographical landscape, promoting open and regular communication, and responsibly exchanging research materials to supporting pan-African research and emphasizing equity and inclusion, these guidelines serve as a blueprint for enhancing Africa’s role in the global research community. They encourage building local capacities, adhering to ethical standards, and embracing open science policies to ensure that research practices are fair and beneficial to all involved. By following these rules, researchers can contribute to a more equitable and dynamic scientific enterprise that not only addresses the challenges faced by the continent but also leverages its unique opportunities for significant global impact.

## References

[1] Bukvova H. Association for Information Systems AIS Electronic Library (AISeL) All Sprouts Content Sprouts 1-14-2010 Studying Research Collaboration. A Literature. Review. 2017.

[2] Haelewaters D, Hofmann TA, Romero-Olivares AL. Ten simple rules for Global North researchers to stop perpetuating helicopter research in the Global South. PLoS Comput Biol. 2021 Aug 19;17(8):e1009277. doi: 10.1371/journal.pcbi.1009277 34411090 PMC8376010

[3] Sonnenwald D. Scientific collaboration. Annu Rev Inf Sci Technol. 2007 Dec 31;41:643–81.

[4] Qin J, Lancaster F, Allen B. Types and Levels of Collaboration in Interdisciplinary Research in the Sciences. JASIS. 1997 Oct 1; 48:893–916.

[5] Liu J, Guo X, Xu S, Zhang Y. Quantifying the impact of strong ties in international scientific research collaboration. PLoS ONE. 2023 Jan 17;18(1):e0280521. doi: 10.1371/journal.pone.0280521 36649356 PMC9844855

[6] Guerrero Bote VP, Olmeda-Gómez C, de Moya-Anegón F. Quantifying the benefits of international scientific collaboration. J Am Soc Inf Sci Technol. 2013;64(2):392–404.

[7] Boekholt P, Cunningham P, Edler J. Drivers of international collaboration in research [Internet]. Publications Office of the European Union; 2009 [cited 2024 Mar 24]. Available from: https://data.europa.eu/doi/10.2777/81914.

[8] Anghileri D, Kandel M, Austen MC, Cheung VV, Coskeran H, Devenish AJM, et al. Rethinking North–South Research Partnerships Amidst Global Uncertainties: Leveraging Lessons Learned from UK GCRF Projects during COVID-19. Land. 2023 Apr;12(4):791.

[9] Vieira ES. International research collaboration in Africa: a bibliometric and thematic analysis. Scientometrics. 2022 May 1; 127(5):2747–72.

[10] Dodsworth S. The Challenges of Making Research Collaboration in Africa More Equitable. In: Oxford Research Encyclopedia of Politics [Internet]. 2019 [cited 2024 Mar 24]. Available from: https://oxfordre.com/politics/display/10.1093/acrefore/9780190228637.001.0001/acrefore-9780190228637-e-1389.

[11] Marincola E, Kariuki T. Quality Research in Africa and Why It Is Important. ACS Omega. 2020 Sep 29;5(38):24155–7. doi: 10.1021/acsomega.0c04327 33015430 PMC7528179

[12] Swartz TH, Palermo AGS, Masur SK, Aberg JA. The Science and Value of Diversity: Closing the Gaps in Our Understanding of Inclusion and Diversity. J Infect Dis. 2019 Aug 20;220(220 Suppl 2):S33–41. doi: 10.1093/infdis/jiz174 31430380 PMC6701939

[13] Minbaeva D, Fitzsimmons S, Brewster C. Beyond the double-edged sword of cultural diversity in teams: Progress, critique, and next steps. J Int Bus Stud. 2021;52(1):45–55. doi: 10.1057/s41267-020-00390-2 33487776 PMC7812974

[14] Demissie B, Nyssen J, Annys S, Negash E, Gebrehiwet T, Abay F, et al. Geospatial solutions for evaluating the impact of the Tigray conflict on farming. Acta Geophys. 2022 Jun 1;70:1285–99.

[15] Sibai AM, Rizk A, Coutts AP, Monzer G, Daoud A, Sullivan R, et al. North-South inequities in research collaboration in humanitarian and conflict contexts. Lancet Lond Engl. 2019 Nov 2;394(10209):1597–600. doi: 10.1016/S0140-6736(19)32482-1 31690433

[16] Asfaha T, Nyssen J, Negash E, Meaza H, Tesfamariam Z, Frankl A, et al. Challenges and resilience of an indigenous farming system during wartime (Tigray, North Ethiopia). Agron Sustain Dev. 2022 Dec 2;42:116.

[17] Negash E, Birhane E, Gebrekirstos A, Gebremedhin MA, Annys S, Rannestad MM, et al. Remote sensing reveals how armed conflict regressed woody vegetation cover and ecosystem restoration efforts in Tigray (Ethiopia). Sci REMOTE Sens [Internet]. 2023 [cited 2024 Mar 24];8. Available from: http://hdl.handle.net/1854/LU-01HG0XA06FEGQXFR778ACSGEHP.

[18] Stahl GK, Maznevski ML. Unraveling the effects of cultural diversity in teams: A retrospective of research on multicultural work groups and an agenda for future research. J Int Bus Stud. 2021 Feb 1;52(1):4–22. doi: 10.1057/s41267-020-00389-9 33487775 PMC7812115

[19] Gervits F, Eberhard K, Scheutz M. Team Communication as a Collaborative Process. Front Robot AI. 2016;3:62. doi: 10.3389/frobt.2016.00062

[20] Morrison-Smith S, Ruiz J. Challenges and barriers in virtual teams: a literature review. SN Appl Sci. 2020 May 20;2(6):1096.

[21] Envuladu EA, Miner CA, Oloruntoba R, Osuagwu UL, Mashige KP, Amiebenomo OM, et al. International Research Collaboration During the Pandemic: Team Formation, Challenges, Strategies and Achievements of the African Translational Research Group. Int J Qual Methods. 2022 Apr 1;21:16094069221115504.

[22] Moseley WG. Collaborating in the field, working for change: Reflecting on partnerships between academics, development organizations and rural communities in Africa. Singap J Trop Geogr. 2007;28(3):334–347.

[23] Boum Ii Y, Burns BF, Siedner M, Mburu Y, Bukusi E, Haberer JE. Advancing equitable global health research partnerships in Africa. BMJ Glob Health. 2018;3(4):e000868. 10.1136/bmjgh-2018-000868 PMC6112391 30167335

[24] Mulder NJ, Adebiyi E, Alami R, Benkahla A, Brandful J, Doumbia S, et al. H3ABioNet, a sustainable pan-African bioinformatics network for human heredity and health in Africa. Genome Res. 2016 Feb;26(2):271–7. doi: 10.1101/gr.196295.115 26627985 PMC4728379

[25] Lauren S. Global Environmental Health Newsletter. [cited 2024 Mar 24]. After 10 Years of Innovation, H3Africa Marks a Milestone. Available from: https://www.niehs.nih.gov/research/programs/geh/geh_newsletter/2022/8/geh-at-niehs/after_10_years_of_innovation_h3africa_marks_a_milestone.

[26] Esterhuizen TM, Li G, Young T, Zeng J, Machekano R, Thabane L. Advancing collaborations in health research and clinical trials in Sub-Saharan Africa: development and implementation of a biostatistical collaboration module in the Masters in Biostatistics Program at Stellenbosch University. Trials. 2021 Jul 22;22(1):478. doi: 10.1186/s13063-021-05427-x 34294129 PMC8295633

[27] African BioGenome Project A. African BioGenome Project–Genomics in the service of conservation and improvement of African biological diversity [Internet]. [cited 2024 Mar 24]. Available from: https://africanbiogenome.org/.

[28] Horn L, Alba S, Gopalakrishna G, Kleinert S, Kombe F, Lavery JLavery, et al. WCRIF—The World Conferences on Research Integrity Foundation. 2024 [cited 2024 Mar 24]. Cape Town Statement. Available from: https://www.wcrif.org/guidance/cape-town-statement.

[29] Parker M, Kingori P. Good and Bad Research Collaborations: Researchers’ Views on Science and Ethics in Global Health Research. PLoS ONE. 2016 Oct 13;11(10):e0163579. doi: 10.1371/journal.pone.0163579 27737006 PMC5063577

[30] Naidoo AV, Hodkinson P, King LL, Wallis LA. African authorship on African papers during the COVID-19 pandemic. BMJ Glob Health. 2021 Mar 1;6(3):e004612. doi: 10.1136/bmjgh-2020-004612 33648979 PMC7925242

[31] Horn L, Alba S, Blom F, Faure M, Flack-Davison E, Gopalakrishna G, et al. Fostering Research Integrity through the promotion of fairness, equity and diversity in research collaborations and contexts: Towards a Cape Town Statement (pre-conference discussion paper). 2022.

[32] Feely AJ, Harzing A. Language management in multinational companies. Cross Cult Manag Int J. 2003 Jun 1;10(2):37–52.

[33] Feitosa J, Hagenbuch S, Patel B, Davis A. Performing in diverse settings: A diversity, equity, and inclusion approach to culture. Int J Cross-cult Manag. 2022;22(3):433–457. doi: 10.1177/14705958221136707

[34] Maponga CC, Mhazo AT, Morse GD. A framework for sustainable capacity-building for collaborative North–South translational health research and training in a resource-constrained setting. Health Res Policy Sys. 2023;21:24. doi: 10.1186/s12961-023-00972-0 36973698 PMC10044759

[35] Sumathipala A, Siribaddana S, Patel V. Under-representation of developing countries in the research literature: ethical issues arising from a survey of five leading medical journals. BMC Med Ethics. 2004 Oct 4;5(1):5. doi: 10.1186/1472-6939-5-5 15461820 PMC524359

[36] Levinson MP. Accountability to research participants: unresolved dilemmas and unravelling ethics. Ethnogr Educ. 2010 Jun;5(2):193–207.

[37] Wilkinson MD, Dumontier M, Aalbersberg IJJ, Appleton G, Axton M, Baak A, et al. The FAIR Guiding Principles for scientific data management and stewardship. Sci Data. 2016 Mar 15;3:160018. doi: 10.1038/sdata.2016.18 26978244 PMC4792175

[38] Kombe F, Anunobi EN, Tshifugula NP, Wassenaar D, Njadingwe D, Mwalukore S, et al. Promoting research integrity in Africa: an African voice of concern on research misconduct and the way forward. Dev World Bioeth. 2014 Dec;14(3):158–66. doi: 10.1111/dewb.12024 23594261 PMC3720696

[39] Kass NE, Hyder AA, Ajuwon A, Appiah-Poku J, Barsdorf N, Elsayed DE, et al. The Structure and Function of Research Ethics Committees in Africa: A Case Study. PLoS Med. 2007 Jan 23;4(1):e3. doi: 10.1371/journal.pmed.0040003 17253898 PMC1779815

[40] Ettarh R. Patterns of international collaboration in cardiovascular research in sub-Saharan Africa. Cardiovasc J Afr. 2016 Jul 6;27(3):194–200. doi: 10.5830/CVJA-2015-082 27841904 PMC5101473

[41] Bhutta ZA, Sommerfeld J, Lassi ZS, Salam RA, Das JK. Global burden, distribution, and interventions for infectious diseases of poverty. Infect Dis Poverty. 2014;3:21. doi: 10.1186/2049-9957-3-21 25110585 PMC4126350

[42] Campbell MC, Tishkoff SA. African genetic diversity: implications for human demographic history, modern human origins, and complex disease mapping. Annu Rev Genomics Hum Genet. 2008;9:403–433. doi: 10.1146/annurev.genom.9.081307.164258 18593304 PMC2953791

